# Antioxidant, antibacterial, enzyme inhibition and fluorescence characteristics of unsymmetrical thiourea derivatives

**DOI:** 10.1016/j.heliyon.2024.e31563

**Published:** 2024-05-21

**Authors:** Faizan Ur Rahman, Abdul Bari Shah, Mian Muhammad, Ezzat khan, Farid S. Ataya, Gaber El-Saber Batiha

**Affiliations:** a Department of Chemistry, University of Malakand, Dir Lower, 18800, Khyber Pakhtunkhwa, Pakistan; b Natural Products Research Institute, College of Pharmacy, Seoul National University, Seoul, 08826, Republic of Korea; c Department of Biochemistry, College of Science, King Saud University, PO Box 2455, Riyadh, 11451, Saudi Arabia; d Department of Pharmacology and Therapeutics, Faculty of Veterinary Medicine, Damanhour University, Damanhour, 22511, AlBeheira, Egypt

**Keywords:** Thiourea derivatives, Unsymmetrical thioureas, Structural characterization, Free radical scavenging, Antimicrobial, Fluorescence characteristics, Enzyme inhibition

## Abstract

A series of six unsymmetrical thiourea derivatives, namely 1-cyclohexyl-3-(pyridin-2-yl) thiourea (**1**), 1-cyclohexyl-3-(3-methylpyridin-2-yl)thiourea (**2**), 1-cyclohexyl-3-(2,4-dimethylphenyl) thiourea (**3**), 1-(4-chlorophenyl)-3-cyclohexylthiourea (**4**), 1-(3-methylpyridin-2-yl)-3-phenylthiourea (**5**), and 1-(3-chlorophenyl)-3-phenylthiourea (**6**), were successfully synthesized via reaction between different amines with isothiocyanates under a non-catalytic environment. Structural elucidation of compounds (**1–6**) was performed using FT-IR and NMR (^1^H and ^13^C) spectroscopy. The infrared spectra displayed characteristic stretching vibrations, while the ^13^C NMR chemical shifts of the thiourea moiety (C=S) were observed in the range of 179.1–181.4 ppm. The antioxidative and antimicrobial properties of the compounds were assessed, as well as their inhibitory effects on acetylcholinesterase and butyrylcholinesterase were evaluated. In order to analyze the fluorescence characteristics of each compound (**1–6**), the excitation (λ_ex_) and emission (λ_em_) wavelengths were scanned within the range of 250–750 nm, with the solvent blank serving as a standard. It was observed that when dissolved in acetone, toluene, tetrahydrofuran, and ethyl acetate, these compounds exhibited emission peaks ranging from 367 to 581 nm and absorption peaks ranging from 275 to 432 nm.

## 1 Introduction

Thiourea derivatives have garnered considerable interest due to their significant role in the realm of nanoparticles, numerous chemical reactions, and extensive coverage in literature [[1], [2], [3], [4], [5]]. The accessibility and efficiency of thiourea derivatives are an ongoing issue in various branches of chemistry [6,7]. These derivatives are currently used in organic synthetic chemistry as building blocks to produce heterocyclic molecules. Their inclination for hydrogen bonding both within and between molecules makes them a desirable model for the study of solid-state chemistry. Moreover, the attractiveness and adaptability of C=S groups within a single molecule are increased for future chemical research. Many scientists are currently exploring the possibilities of this adaptable class of substances [8]. In commercial, industrial, and academic contexts, these derivatives hold significant importance and play an active role. Various thiourea derivatives such as cyclohexyl and phenyl are employed in the pharmaceutical industry as promising therapeutic agents, exhibiting properties such as anti-HIV, anti-HCV [[9], [10], [11]], anticancer, anticonvulsant [12,13], antihyperlipidemic, antiallergic, antiparasitic, antiproliferative, antioxidant, and antidiabetic effects [14,15]. Derivatives of thiourea have been extensively studied, highlighting the significant consequences of this diverse class of chemicals in a range of industrial and environmental settings. The study has illuminated the various uses of thiourea derivatives and shown their applicability and potential influence in a range of domains [[16], [17], [18], [19], [20], [21]]. Presently, one important, diverse, and effective application of thioureas lies in their role as organocatalysts in Suzuki and Heck reactions, as these compounds exhibit thermal stability and enables reactions to be conducted under ambient conditions [22].

Reactive oxygen species (ROS), or free radicals, are generated during instances of oxidative stress. These substances have been identified as putative initiators of several clinical illnesses, such as neurological diseases, diabetes, atherosclerosis, and cancer [23]. Similarly, it has been determined that the overexpression of butyryl and acetylcholinesterase contributes to the development of neurodegenerative disorders [24,25]. Conversely, bacterial infections have been linked to a variety of illnesses [26]. That being said, in order to address these health issues in the human population, it is imperative that strong substances be identified and used as supplements. In addition to various other applications, thiourea derivatives exhibit remarkable sensing capabilities for a wide range of metal ions. Fluorescence, an essential physical phenomenon, involves the emission of electromagnetic radiation of longer wavelengths by a specific chemical compound (such as thiourea) when excited by shorter-wavelength radiation. One of the many advantages of fluorescence technology is its ability to detect single molecules. This feature makes high throughput screening and sample size reduction easier. Given that mercury is one of the most dangerous elements that is frequently present in soil and water [[27], [28], [29], [30], [31], [32]]. Literature reports indicate that thiourea derivatives demonstrate absorption maxima ranging from 275 to 432 nm and emission maxima ranging from 367 to 581 nm in solvents such as acetone, toluene, tetrahydrofuran, and ethyl acetate [33].

Fluorescence is widely recognized as a highly versatile technique with a wide range of applications, making it a preferred choice for detecting environmental samples and for microscopy and imaging purposes [34]. Its exceptional sensitivity and specificity render it highly important in the fields of environmental monitoring, pharmaceutical analysis, and forensic science. Carefully designed fluorescent probes can be employed to selectively target specific analytes, thereby enhancing their detectability [35]. It can be utilized for the objective of observing environmental features, containing pH, metal ion concentrations, and levels of pollutants, with a main focus on these features affecting human health in the environment [36]. The distinctive property of thiourea derivatives to detect both cations and anions renders them exceptionally adaptable detectors that are anticipated to garner considerable interest in the coming years [5]. Thiourea derivatives have the capability to identify heavy metal ions (e.g., mercury ions (Hg^2+^) and other anions) in water-based systems through fluorescence because of its strong affinity and bonding. Because of the functional group of thiourea, the Hg^2+^ and thiourea derivatives are attracted towards each other, resulting in coordinate covalent bond. Thiourea derivatives can react with mercury ions to produce stable complexes, resulting in noticeable alterations in characteristics such as color or fluorescence. This interaction serves as the foundation for multiple analytical procedures used to identify and measure the amount of mercury in solutions [[37], [38], [39]].

**Fig. 1:**
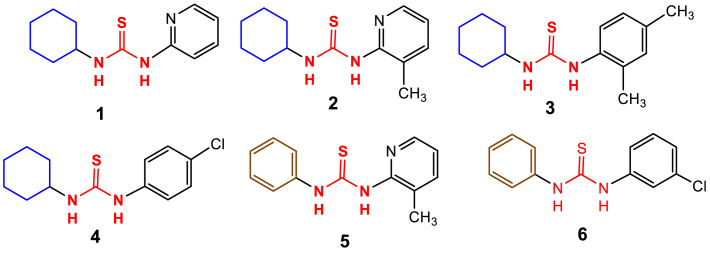
Structure of synthesized compounds (**1–6**) used in this study.

This study focuses on the biological screening of unsymmetrical cyclohexyl and phenylthiourea derivatives with fluorescent properties, as illustrated in (Fig. 1). A thorough analysis was performed on each synthesized molecule to determine its butyryl and acetylcholinesterase, antibacterial, and antioxidant properties. Their potential as physiologically active compounds with attributes including antioxidant, antibacterial, and enzyme inhibitory activities was the focus of the investigation. These substances showed promising biological activity and were found to be excellent enzyme inhibitors, strong antioxidants, and powerful antimicrobials. These chemicals also exhibit potential as environmental sensing agents, namely for identifying metal ions, potent insecticides, and pharmaceuticals.

## 2 Materials and methods

### 2.1 General overview and synthesis

The reactions were conducted under normal atmospheric conditions, without any deliberate efforts to remove air or moisture during the experimental processes. Spectroscopic techniques, specifically ATR-FTIR spectroscopy, in conjunction with proton (1H) and carbon-13 (13C) NMR spectroscopy, were employed to validate the structures of the compound. The evaluation of the enzyme inhibition solution absorption was conducted by Thermoelectron Corporation, USA. Additionally, fluorescence experiments were conducted using the RF-5301 Shimadzu fluorescence spectrophotometer model from Japan. These studies were performed to assess their potential application in detecting environmental pollutants. According to the previous literature, thiourea derivatives were prepared [[40], [41], [42], [43]] as, by dissolving 0.6 g (7.05 mmol) of 2-aminopyridine in 20 mL of acetone. Afterwards, a solution of cyclohexylisothiocyanate (1 mL, 7.05 mmol) was slowly added to the mixture. The resultant reaction mixture was agitated for an extended period of time at the ambient temperature. Precipitates were formed during the reaction, and their development was tracked using Thin Layer Chromatography (TLC). The solid material that formed was isolated from the liquid solution, dissolved in ethanol, and then allowed to evaporate slowly. The structure of the target molecule was determined by acquiring and analyzing spectroscopic data. The remaining compounds are presented in supplementary materials.

### 2.2 Free radical scavenging assay

In accordance with the procedure described in the literature [23,44], the ABTS activity assay was applied to evaluate the free radical scavenging capabilities of the synthesized compounds (**1–6**). ABTS solution was prepared by mixing 7 mM ABTS and 2.45 mM potassium persulfate and was then placed for 16 h under dark at room temperature. A methanol dilution was performed on the solution to achieve an absorbance of 0.706 ± 0.001 at 734 nm before starting the assay. To quantify the assay, 3 mL of the ABTS solution has been added to different concentrations (ranging from 62.5 to 1000 μg/mL) of every compound (300 μL) that were dissolved in methanol. Then after 1 min of incubation, the absorbance was continuously measured for 5 min. To confirm precision, all experiments were conducted in triplicate. The antioxidant capacities of the compounds were assessed with gallic acid as a reference, and the ability to remove free radicals was calculated as a percentage for each compound employing Equation.1 as presented below. Additionally, the DPPH free radical scavenging method was employed to assess the antioxidant activity of compounds (**1–6**), in accordance with a widely recognized procedure documented in existing literature [45]. Concentrations ranging from 62.5 to 1000 μg/mL of compounds (**1–6**), dissolved in methanol, were introduced into a 2 mL solution of DPPH (0.002 % in methanol). The resulting solutions were kept in the dark at room temperature for a duration of 30 min. The amount of light absorbed by the solutions was then determined using a UV/vis spectrophotometer at a wavelength of 517 nm. The investigations were performed three times, using the exact protocol described for the ABTS scavenging assay. Afterwards, the antioxidant capabilities of each molecule were assessed using calculation.

(1) $$\text{Scavenging}\;\text{activity}\;\left(\%\right)=\frac{A-B}{A}\times 100$$

Where, A = absorbance of ABTS/DPPH and B = absorbance of solution containing ABTS/DPPH and test samples.

### 2.3 Antibacterial assay

The antibacterial assay was done for all compounds (**1–6**) as described in previous studies [46,47]. The mean number of viable organisms per milliliter of stock suspensions containing *Escherichia coli* (ATCC25922), *Pseudomonas aeruginosa* (ATCC27853), *Salmonella typhi* (ATCC0650), and *Staphylococcus aureus* (NCTC25953) was uncovered using the surface viable counting method. Preparations of suspensions containing approximately 108–109 colony forming units (CFU) per milliliter were made. Aseptic nutritional agar was formulated and transferred onto petri dishes. Cultures of bacteria were uniformly distributed on agar surface using sterile brush sticks. Five wells, each with a diameter of 6 mm, were made in each plate using a sterile borer. Afterwards, a concentration of 6.25 mg/mL of each component was applied to separate wells. Cephradine, a readily accessible antibiotic, was employed as a reference standard at an equivalent concentration. Following sealing, the agar dishes were incubated at a controlled temperature of 37 °C for a period of 24 h. The zone of inhibition in the plates was determined, which represents the inhibition of bacterial growth. Each sample zone of inhibition diameter was evaluated, and the antibacterial effectiveness of each drug was quantified according to the mean diameter of the inhibition zone (in millimetres).

### 2.4 Anticholinesterase assay

Elman's test was applied to estimate the enzyme inhibitory activity of the synthesized thiourea derivatives (**1–6**). This assay employed two enzymes, namely acetylcholinesterase (AChE) and butyryl cholinesterase (BChE) [48,49]. The AChE was derived from the *electric eel*, while BChE was obtained from *equineserum*. The synthesized compounds were dissolved in phosphate buffer (0.1 M, pH 8.0). The enzymes AChE (518 U/mg) and BChE (7–16 U/mg) were diluted in a 0.1 M phosphate buffer to achieve concentrations of 0.03 U/mL and 0.01 U/mL, respectively. Preparations of ATchI (0.5 mM), DTNB (0.2273 mM), and BTchI (0.5 mM) were made in distilled water and stored at a low temperature of 8 °C. In a cuvette, 5 μL of the enzyme solution, 206 ± 1 μL of the test compounds (**1–6**), and 5 ± 1 μL of the DTNB reagent were combined. The solution was heated to 30 °C for 15 min, followed by the gradual addition of the substrate. Absorbance of the heated sample at 412 nm was measured using a double beam spectrophotometer. Galantamine was utilized as a positive control in each experiment, which was performed in triplicate. The percentage activity (A) and inhibition of the enzyme were determined by applying the rate of absorption as a function of time $\left(\;V=\Delta_{Abs/}\Delta_{t\;}\right)$ using the following equation (Equation (2)).

(2) $$\%\text{Inhibition}=\frac{V}{V_{\max}}\times 100$$

Where V = enzyme inhibition activity of products and V_max_ = enzyme activity without test of product.

### 2.5 Fluorescence measurements

The fluorescence measurements were conducted using a Shimadzu RF-5301 PC spectrofluorophotometer, which was fitted with a 150-W Xenon lamp as the excitation source and a 1.0 cm quartz cell. The fluorimetric operation employed emission and excitation slits of 4 nm. The thiourea derivatives were prepared at varying concentrations in acetone and incubated for a duration of 60 min at room temperature. Notably, compounds **1–6** exhibited excellent fluorescence behavior at both low and high sensitivity levels. Among the thiourea derivatives, compound **3** was employed in the creating of a sensing instrument intended for the examination of mercury levels in water samples.

### 2.6 Investigation of the interaction of compounds (1–6) with Hg (II)

Compounds (**1–6**) at a concentration of 50 μg mL-1 and Hg^2+^ at a concentration of 10 μg mL^−1^ as HgCl_2_ was prepared in 10 mL volumetric flasks. Afterwards, the mixture was diluted with distilled water until it reached the specified level, and the fluorescence intensity (FI) was measured for each sample in comparison to the reagent blanks.

### 2.7 Investigation of the interaction of metal ions with compound 3

The experimental procedure consisted of mixing 1.0 mL of compound **3,** which had a concentration of 50 μg/mL, with 1.0 mL of different metal ions, each at a concentration of 1.0 μg/mL. These include Hg^2+^ as HgCl_2_, Mn^2+^, Co^2+^, Mg^2+^, Zn^2+^, Cd^2+^, Ca^2+^, Ni^2+^ and Cu^2+^, in a series of 10 mL volumetric flasks. Afterwards, the mixture was diluted with distilled water up to the designated level, and the FI was determined for each combination in comparison to the reagent blanks.

## 3 Results and discussion

Compounds (**1–6**) possess a simple structure and exhibit strong biological effects. These compounds can be readily synthesized in the laboratory under normal environmental conditions [[50], [51], [52]]. Compounds that have a similar structure to (**1–6)** are effective corrosion inhibitors in an acidic environment [53] and demonstrate promising results in utilizing metal sulfides for practical applications [54]. The usefulness of modified simple thiourea molecules as bioactive substances has been documented in a number of additional investigations [55]. The inhibitory action of Compounds **5** and **6** against melanin B16 cells and mushroom tyrosinase were checked previously, and their preparation has been reviewed [25].

### 3.1 Free radical scavenging essay

Free radicals are substances that are reactive to chemicals and mostly consist of unpaired electrons. The human body constantly produces them, and the body defense mechanism quickly scavenges them. However, external antioxidant supplementation is necessary when the body cannot produce them all by itself. If not, they can harm components that are crucial to biology, such proteins and DNA. All the synthesized compounds in this work exhibited scavenging capabilities against commercially available free radicals, namely DPPH and ABTS, as indicated in Table 1. Compound **5** exhibited the highest potency against DPPH free radical (IC_50_ = 37.50 μg/mL), and subsequently compound **1** (IC_50_ = 63.02 μg/mL). Compounds **4**, **2**, and **6** also displayed significant efficacy in scavenging the DPPH radical, with IC_50_ values of 67.11, 72.00, and 90.30 μg/mL, respectively. Compound **3** exhibited the lowest effectiveness with an IC_50_ value of 118.05 μg/mL. Similar inhibitory patterns were observed for the ABTS free radical. Compound **5** demonstrated the highest potency with an IC_50_ value of 44.60 μg/mL, followed by compound **1** (IC_50_ = 67.08 μg/mL). Compounds **4**, **2**, and **6** showed moderate effectiveness, while compound **3** displayed the least.

**Table 1:** Antioxidant efficiency of compounds (**1–6**) against DPPH and ABTS free radicals.

Compound	Conc. (μg/mL)	DPPH	IC_50_	ABTS	IC_50_(μg/mL)
Number		%inhibition (μg/mL)	(μg/mL)	%inhibition (μg/mL)	
**1**	1000	85.10 ± 1.45	63.02	84.81 ± 1.17	67.08
	500	75.63 ± 2.60		72.20 ± 1.90	
	250	63.01 ± 1.65		63.59 ± 2.50	
	125	54.32 ± 1.89		54.45 ± 0.96	
	62.5	48.57 ± 2.03		47.65 ± 1.74	
**2**	1000	81.22 ± 2.89	72.00	79.87 ± 2.65	82.02
	500	73.41 ± 2.87		70.37 ± 2.37	
	250	64.15 ± 2.10		60.39 ± 1.71	
	125	54.86 ± 2.86		51.10 ± 1.39	
	62.5	46.51 ± 2.74		45.52 ± 2.80	
**3**	1000	80.38 ± 1.10	118.05	80.11 ± 1.01	115.07
	500	71.19 ± 1.27		71.16 ± 2.10	
	250	62.53 ± 1.11		62.14 ± 1.30	
	125	51.88 ± 1.29		51 0.40 ± 1.44	
	62.5	46.72 ± 2.13		46.06 ± 1.86	
**4**	1000	82.11 ± 1.20	67.11	81.23 ± 1.01	67.01
	500	72.29 ± 0.27		71.03 ± 1.61	
	250	63.74 ± 0.81		61.14 ± 1.43	
	125	54.67 ± 2.08		56 0.11 ± 1.71	
	62.5	48.18 ± 2.31		48.19 ± 1.26	
**5**	1000	88.58 ± 1.12	37.50	92.76 ± 0.71	44.60
	500	81.65 ± 1.34		85.23 ± 1.83	
	250	74.31 ± 2.15		77.42 ± 0.43	
	125	67.56 ± 1.73		72.56 ± 1.06	
	62.5	62.44 ± 0.58		67.80 ± 1.50	
**6**	1000	82.19 ± 2.32	90.30	81.28 ± 2.17	86.03
	500	76.24 ± 2.61		75.19 ± 2.59	
	250	61.47 ± 2.74		63.09 ± 1.34	
	125	53.66 ± 1.17		53.26 ± 2.76	
	62.5	43.49 ± 2.72		44.27 ± 1.53	
**Gallic acid**	1000	81.45 ± 0.65	27.06	75.89 ± 0.20	39.54
	500	75.45 ± 0.45		71.88 ± 0.20	
	250	69.47 ± 0.46		66.43 ± 0.29	
	125	63.83 ± 1.07		59.84 ± 0.32	
	62.5	57.54 ± 0.46		51.68 ± 0.22	

### 3.2 Antibacterial activity

The synthesized compounds (**1–6**) were tested for their antimicrobial activity using the agar well diffusion technique. The antibacterial potentials of these compounds were tested against selected strains of bacteria, including *E. coli*, *S. flexenari*, *P. aeruginosa*, and *S. typhi*. Although none of the stated compounds exhibited strong activity, they demonstrated moderate antibacterial effects compared to the standard antibacterial drug Cephradine. Inhibition zones for these compounds against the tested bacterial strains varied from 8 to 15 mm. Among the compounds, **1** exhibited superior antibacterial activity against *S. flexneri*, while **2** was more effective against *E. coli* and *S. typhi*. Compound **3** demonstrated activity against *E. coli* comparable to that of the standard drug. Compound **4** showed the highest activity against both *S. flexneri* and *P. aeruginosa* among all tested compounds. Similarly, **5** and **6** exhibited the highest activity against *S. flexneri* and *E. coli*, respectively, as shown in Table 2.

**Table 2:** Antimicrobial activities of compound 1-6.

Compound	Bacteria	Zone of inhibition of sample(mm)	Zone of inhibition of Std.Drug (mm
**1**	*E. coli*	9.5	30
	*S. typhi*	10.0	25
	*S. flexenari*	**8.5**	**12**
	*P. aeruginosa*	9.0	35
**2**	*E. coli*	**9.5**	**11**
	*S. typhi*	**11.5**	**13**
	*S. flexenari*	11.0	29
	*P. aeruginosa*	13.5	30
**3**	*E. coli*	**11.0**	**12**
	*S. typhi*	8.5	15
	*S. flexenari*	10.0	12
	*P. aeruginosa*	13.5	29
**4**	*E. coli*	12.0	25
	*S. typhi*	11.5	25
	*S. flexenari*	**13.0**	**15**
	*P. aeruginosa*	**10.0**	**9**
**5**	*E. coli*	10.5	25
	*S. typhi*	11.5	20
	*S. flexenari*	**10.5**	**15**
	*P. aeruginosa*	9.5	31
**6**	*E. coli*	**8.5**	**10**
	*S. typhi*	9.5	14
	*S. flexenari*	10.5	13
	*P. aeruginosa*	15.5	28

### 3.3 Enzyme inhibition studies

The inhibitory potential of all compounds **(1–6**) against the selected cholinesterases was evaluated. The results of these studies are summarized in Table 3. Most of the compounds exhibited high IC_50_ values (>100 μg/mL), except for two compounds. Compound **6** demonstrated inhibitory activity below 100 μg/mL, while compound **1** exhibited the most potent inhibition with reasonable activity at 27.05 and 22.60 μg/mL against AChE and BChE, respectively. The IC_50_ value for the standard was 19.67 μg/mL and 17.89 μg/mL for the respective inhibition of these enzymes. Future studies may involve further derivatization of compound **1** to achieve the desired efficacy.

**Table 3:** Enzyme inhibition effectiveness of compounds (**1–6**) against AChE and BChE.

Compound name	Concentration (μg/mL)	AChE Percent inhibition	IC_50_	BChE Percent inhibition	IC_50_
			(μg/mL)		(μg/mL)
**1**	1000	88.58 ± 1.12	27.5	91.76 ± 0.71	22.60
	500	81.65 ± 1.34		84.23 ± 1.83	
	250	74.31 ± 2.15		76.42 ± 0.43	
	125	67.56 ± 1.73		71.56 ± 1.06	
	62.5	62.44 ± 0.58		65.80 ± 1.50	
**2**	1000	62.61 ± 0.77	369.86	64.79 ± 0.62	314.78
	500	54.60 ± 0.80		56.45 ± 0.49	
	250	43.83 ± 0.56		45.75 ± 0.58	
	125	35.69 ± 0.77		37.51 ± 0.77	
	62.5	29.67 ± 0.61		31.53 ± 0.71	
**3**	1000	65.50 ± 2.26	143.76	67.44 ± 0.09	122.79
	500	59.01 ± 0.42		61.87 ± 0.39	
	250	53.07 ± 0.62		55.83 ± 1.07	
	125	49.70 ± 0.35		50.23 ± 0.44	
	62.5	43.73 ± 0.66		44.29 ± 0.43	
**4**	1000	69.58 ± 1.12	249.57	71.33 ± 0.49	218.83
	500	61.65 ± 1.34		63.03 ± 0.23	
	250	47.90 ± 0.96		49.00 ± 0.58	
	125	39.03 ± 0.48		42.67 ± 0.89	
	62.5	31.90 ± 0.48		33.00 ± 1.15	
**5**	1000	63.34 ± 0.98	342.34	69.58 ± 1.12	249.57
	500	56.32 ± 1.06		61.65 ± 1.34	
	250	48.05 ± 0.75		47.90 ± 0.96	
	125	44.70 ± 1.25		39.03 ± 0.48	
	62.5	38.74 ± 0.68		31.90 ± 0.48	
**6**	1000	67.51 ± 0.54	86.40	71.60 ± 1.63	56.85
	500	62.84 ± 0.30		66.80 ± 7.85	
	250	57.80 ± 1.50		61.25 ± 1.40	
	125	52.72 ± 1.01		55.10 ± 0.60	
	62.5	47.61 ± 0.43		51.61 ± 0.43	
**Galantamine**	1000	83.19 ± 0.73	19.67	89.64 ± 0.62	17.89
	500	77.54 ± 0.91		81.83 ± 0.37	
	250	71.93 ± 0.14		74.29 ± 0.73	
	125	65.72 ± 0.49		67.92 ± 0.98	
	62.5	61.67 ± 0.94		64.93 ± 0.87	

### 3.4 Fluorescence characteristics

**Fig. 2:**
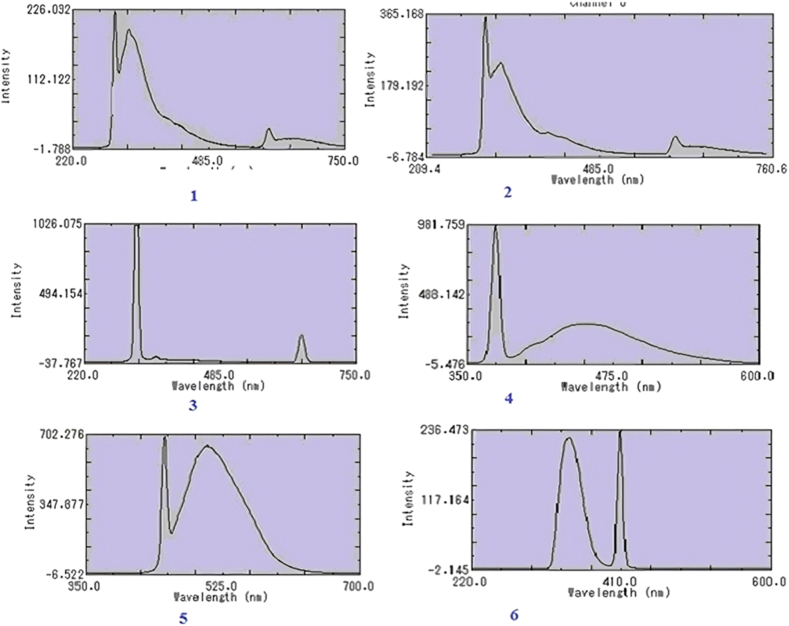
Fluorescence spectra of compounds. Each compound is represented by their number from **1**–**6**.

To examine the fluorescence properties of compounds (**1–6**), λ_ex_ and λ_em_ were scanned against a solvent blank within the wavelength range of 250–750 nm. The fluorescence spectra of the compounds are depicted in (Fig. 2), and the corresponding data are presented in Table 4. Notably, compound **1, 5**, and **6** exhibited excellent fluorescence intensity (FI) at low sensitivity levels, while compounds **2–4** displayed remarkable FI at high instrumental sensitivity.

**Table 4:** Fluorescence properties of compounds (**1–6)**, measured in nm.

Compound	Concentration (μg mL^−1^)	Scan range	λ_ex_	λ_em_	Sensitivity	Fluorescence Intensity
1	10	220–600	348.0	168.00	Low	492.670
2		350–700	451.0	505.00	High	646.576
3		220–750	302.0	223.79	High	193.641
4		350–600	373.0	911.28	High	279.409
5		220–650	346.0	400.00	Low	296.188
6		220–600	343.0	408.00	Low	234.134

The fluorescence behavior of compounds (**1–6**) suggests that even at low concentrations as low as 10 μg/mL, these compounds can be used to develop fluorescence-based sensing systems for identifying environmental pollutants, specifically hazardous metal, pesticides, and pharmaceuticals. The analyte can efficiently engage with the compounds, resulting in either an increase or decrease in fluorescence. As a result, there is potential for developing subtle analytical techniques to identify target analytes by utilizing this interaction.

### 3.5 Application of compound 3 as sensing probe for determination of mercury

**Fig. 3:**
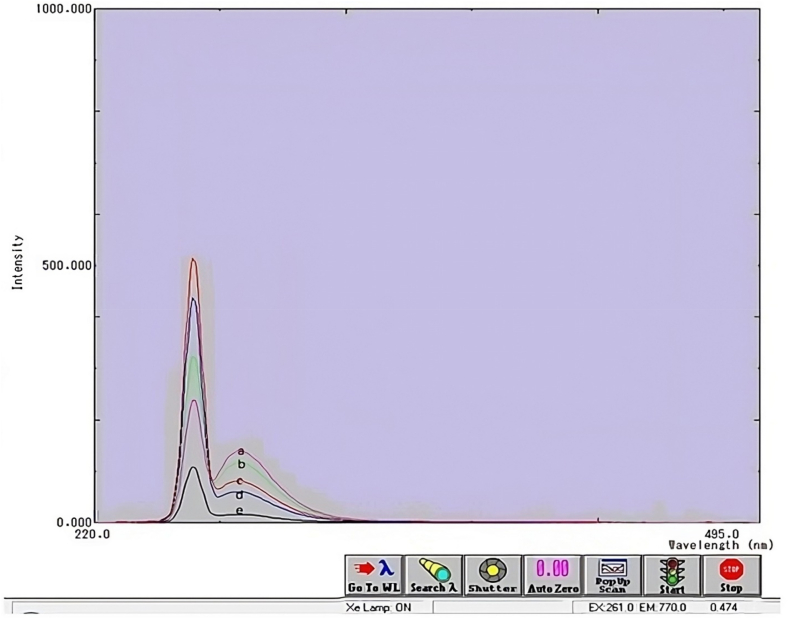
Fluorescence intensity of F16 as a function of the concentration of Hg(II); Curves e:no Hg(II) added, Curve d, c, b, a:0.01, 0.02, 0.03, 0.04 (μg mL^−1^) Hg(II) added.

Fluorescence intensity of 0.01 μg/mL 1-Cyclohexyl-3-(2,4-dimethylphenyl)thiourea was measured against solvent blank and against variable concentration of Hg(II) solutions in the concentration range of 0.01–0.04 μg/mL with excitation wavelength of 264 nm followed by emission at 284 nm. As evident from the data in given in Table 5 and shown in (Fig. 3), the fluorescence intensity of fixed concentration of 1-Cyclohexyl-3-(2,4-dimethylphenyl)thiourea linearly rises when concentration of Hg(II) increases. The application of this fluorescence enhancement phenomenon has proven to be effective in the quantification of Hg(II) at the trace level in various complex environmental samples, such as fruits, water, and soil [50].

**Table 5:** Fluorescence intensity of 1-Cyclohexyl-3-(2,4-dimethylphenyl)thiourea as a function of the concentration of Hg(II).

Concentration of 3 (μg mL^−1^)	Fluorescence Intensity at λ_em_ = 284 nm	Concentration of Hg(II) (μg mL^−1^)	Fluorescence Intensity at λ_em_ = 284 nm
0.01	16.189 Curve:e	0.01	60.193
		0.02	81.624
		0.03	117.136
		0.04	139.836

Compound **3**, 1-Cyclohexyl-3-(2,4-dimethylphenyl)thiourea, commonly referred to as DMDTU, is frequently utilized in fluorescence detection applications for the determination of mercury detection. Due its chemical and structrual properties it can used for such purpose, which resulting in a specific fluorescence signal because of interaction between mercury ions and thiourea deriviteves. With its distinctive atomic arrangement and functional groups, DMDTU demonstrates an exceptional attraction to mercury ions. With the cyclohexyl and 2,4-dimethylphenyl groups present in DMDTU, its three-dimensional structure is enhanced, allowing for optimal interaction with mercury ions. Thanks to its chelating ability, DMDTU is capable of forming stable complexes with metal ions like mercury, making it a dependable chelating agent. The different characterisicitcs of fluoresence results in evident change when mercurcry ions come in contact with such compound, and thus it results in finding the mercury ions in a simple which is consistent way for such detection. Some of the thiourea derivitives might not have such properties which results in reducing the power of detection of mercury through fluoresence. Based on such properties DMDTU is a perfect choice for mercury detection [25].

### 3.6 Investigation of the interaction of compounds 1–6 with Hg(II)

**Fig. 4:**
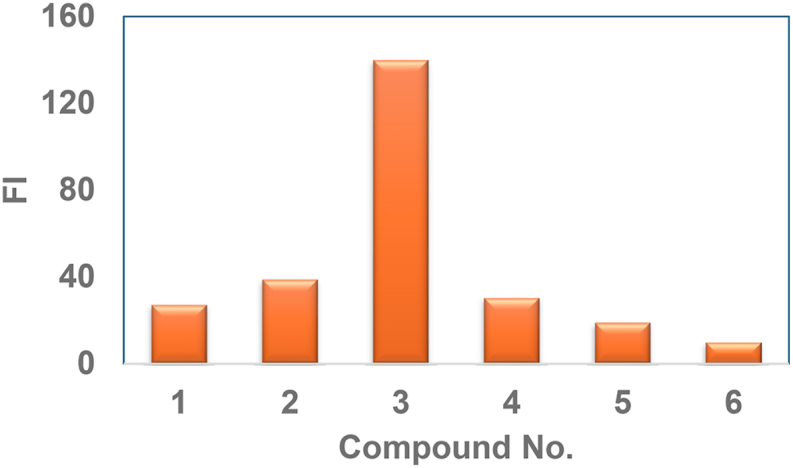
Investigation of the interaction of Compounds (**1–6**) with Hg(II).

Compounds (**1–6**) were reacted with constant concentration of Hg(II) (1.0 μg/mL) in different ratios and the enhancement in FI with compound **3** was found to be significant as compared to the rest of the compounds where the enhancement though high was not enough to be employed for determination of Hg(II) at lower concentration. The results are shown in (Fig. 4).

### 3.7 Investigation of the interaction of metal ions with compound 3

**Fig. 5:**
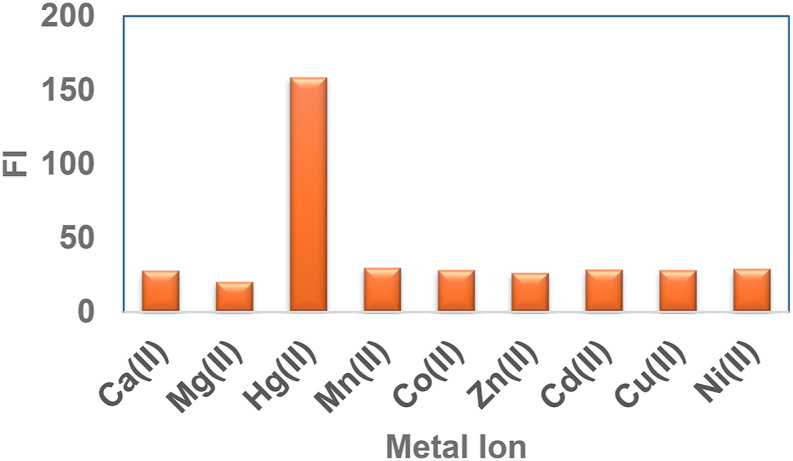
Investigation of the interaction of metal ions with compound 3.

An investigation was conducted on the FI of the Hg^2+^-L3 complex in water samples to determine the effect of various metal ions, including Hg^2+^ as HgCl_2_, Mn^2+^, Co^2+^, Mg^2+^, Zn^2+^, Cd^2+^, Ca^2+^, Ni^2+^ and Cu^2+^ ions (Fig. 5). illustrates the experimental results, which demonstrate that these species exhibit minimal interference.

## 4 Conclusion

Thiourea derivatives (**1–6**) were successfully synthesized without observing any side reactions. The compounds were structurally characterized and assessed for their enzyme inhibitory efficiency. The activity of 1-Cyclohexyl-3-(pyridin-2-yl)thiourea (IC_50_ = 27.05 and 22.60 μg/mL against AChE and BChE, respectively) closely approached the standard drug and warrants tartgeted efficiency in future studies. All of the synthesized compounds exhibited scavenging abilities against commercially available DPPH and ABTS radicals. Compound 1-(3-Methylpyridin-2-yl)-3-phenylthiourea displayed higher potency as an inhibitor of DPPH and ABTS radicals (IC_50_37.50 μg/mL and 44.60 μg/mL, respectively), while compound 1-Cyclohexyl-3-(2,4-dimethylphenyl)thiourea showed the least effectiveness, IC_50_118.05 μg/mL and 115.07 μg/mL, respectively. The 1-Cyclohexyl-3-(2,4-dimethylphenyl)thiourea was also verified as a sensing tool for the determination of mercury. The fluorescence intensity of the compound increased linearly with the concentration of Hg(II) until reaching the maximum fluorescence intensity level. The interaction of the compounds with Hg(II) can be explained using the soft-hard acid-base concept. This fluorescence enhancement can be effectively utilized to accurately detect Hg(II) at low concentrations in various complex environmental samples, such as water, soil, vegetables, and fruits.

## Funding Section

The authors would like to extend their gratitude to King Saud University (Riyadh, Saudi Arabia) for funding this research through Researchers supporting Project number (RSPD2024R693)

## Data availability statement

Data will be made available on request.

## CRediT authorship contribution statement

**Faizan Ur Rahman:** Writing – original draft, Investigation, Data curation, Conceptualization. **Abdul Bari Shah:** Writing – review & editing, Writing – original draft, Supervision, Methodology, Investigation. **Mian Muhammad:** Formal analysis, Conceptualization. **Ezzat khan:** Writing – review & editing, Conceptualization. **Farid S. Ataya:** Funding acquisition, Formal analysis. **Gaber El-Saber Batiha:** Formal analysis, Conceptualization.

## Declaration of competing interest

The authors declare that they have no known competing financial interests or personal relationships that could have appeared to influence the work reported in this paper.
