# Supplementary material for: Antioxidant, antibacterial, enzyme inhibition and fluorescence characteristics of unsymmetrical thiourea derivatives
Source: Heliyon. 2024 May 21;10(10):e31563. doi: 10.1016/j.heliyon.2024.e31563 (PMC11141368; doi:10.1016/j.heliyon.2024.e31563)
Supplement: Multimedia component 1 [file mmc1.docx]

**Supplementary materials**

**Antioxidant, Antibacterial, Enzyme Inhibition and Fluorescence Characteristics of Unsymmetrical Thiourea Derivatives**

Faizan Ur Rahman ^a^, Abdul Bari Shah ^b,^*, Mian Muhammad ^a^, Ezzat khan ^a^, Farid S. Ataya ^c^, Gaber El-Saber Batiha ^d^

*^a^Department of Chemistry, University of Malakand, Dir Lower 18800, Khyber Pakhtunkhwa, Pakistan.*

*^b^Natural Products Research Institute, College of Pharmacy, Seoul National University, Seoul 08826, Republic of Korea*

*^c^Department of Biochemistry, College of Science, King Saud University, PO Box 2455, Riyadh 11451, Saudi Arabia*

*^d^Department of Pharmacology and Therapeutics, Faculty of Veterinary Medicine, Damanhour University, Damanhour 22511, AlBeheira, Egypt*

**Correspondence to:**

Dr. Abdul Bari Shah, Natural Products Research Institute, College of Pharmacy, Seoul National University, Seoul 08826, Republic of Korea

E-mail: [abs.uom28@gmail.com](mailto:abs.uom28@gmail.com), [abs.snu28@snu.ac.kr](mailto:abs.snu28@snu.ac.kr)

**Experimental**

**Physical measurements**

All reactions conducted in this study were performed in a non-catalytic environment, under aerobic conditions, without any specific measures taken to exclude air or moisture during the experiments. The handling of chemicals utilized in this investigation was carried out in an open-air setting. Phenylisothiocyanate, cyclohexylisothiocyanate, 2-aminopyridine, 2-amino-3-methylpyridine, 3-chloroaniline, 4-chloroaniline, and 2,4-dimethylamine (commercial products obtained from Sigma Aldrich) were used as received. Vibrational behavior of molecules in the range of 4000-400 cm-1 was measured using an FT-IR spectrometer employing the ATR sampling technique. 1H and 13C NMR spectra were obtained using a Bruker AM 300 spectrometer (Bruker, Karlsruhe, Germany). The UV/Vis spectrophotometer (Thermo electron, USA) was employed to monitor the antioxidant and enzyme inhibition potentials through UV/Vis measurements. Fluorescence studies were conducted using a fluorescence spectrophotometer RF-5301 to explore the potential of these compounds for sensing environmental pollutants. Thiourea derivatives were prepared via a straightforward reaction without the need for costly catalysts, with slight modifications to our previously reported procedures.

**Synthesis of Compounds 1-6**

**Compound 1:** Yield = 71 %; Molecular formula = C12H17N3S; m.p. 132-134˚C; FT-IR (ATR): ν (cm-1) =3433br (NH), 3217br (N-H), 2930s, 2853w (CH), 1558 (C=S), 1315 (NCN), 1430 (CSasy); 1H-NMR (300 MHz, DMSO-d6) δ (ppm)= 1.20-2.01 (m, 10H, CH2), 2.50 (quentate, 1H, CH), 4.19 (s, 1H, NH), 10.46 (s, 1H, NH), 6.98, 7.17, 7.74, 8.21 (m, m, m, 4H, Ph); 13C-NMR (75 MHz, DMSO-d6) δ (ppm) = 23.1 (2C, CH2), 31.9 (1C, CH2), 52.7 (2C CH2), 53.3 (1C, CH) (Cyclohexyl carbons), 113.1 (1C) 117.7 (1C), 138.9 (1C), 145.6 (1C), 154.6 (1C), (Ph, Carbons), 178.5 (C=S).

**1-Cyclohexyl-3-(3-methylpyridin-2-yl)thiourea, 2**

In the similar manner as described above compound 2 was prepared by mixing 2-anino-3-methylpyridine (0.7mL, 7.05mmol) with a solution of cyclohexylisothiocyante (1.0 mL, 7.05 mmol) in dry acetone. Compound 2 was obtained as a colorless solid in ethanol.

Yield = 70 %; Molecular formula = C13H19 N3S; m.p.135-138°C;FT-IR (ATR) : ν(cm-1) = 3458br, 3244br (N-H), 3070w, 2927w (C-H), 1759s (C=S), 1381 (NCN), 1561 (CSasy); 1H-NMR(300 MHz DMSO d6) δ (ppm) = 0.86, 1.38, 1.64, 1.93, 2.45, 2.50 (q, 11H, Cyclohexyl), 2.27 (s, 3H, Me), 4.19 (s, 1H, NH), 8.55 (s, 1H, NH), 7.02, 7.65, 8.12, 11.85 (d, d, d, d, 3H, Ph); 13C-NMR (75 MHz, DMSO-d6) δ (ppm) = 16.9 (2C, CH2), 24.4 (2C, CH2), 25.5 (2C CH2), (Cyclohexyl carbons) 31.8 (1C, Me), 118.7 (1C) 121.2 (1C), 140.3 (1C), 143.6 (1C), 152.1 (1C), (Ph, Carbons), 178.4 (C=S).

**1-Cyclohexyl-3-(2,4-dimethylphenyl)thiourea, 3**

A known amount of 2,4-dimethylaniline (0.88 mL, 7.05 mmol) in 15 mL analytical grade acetone and equimolar amount of Cyclohexylisothiocynate (1mL, 7.05 mmol) was reacted together following the same method. Colorless crystals of 3 were obtained in EtOH in few days, were separated from the mother liquor, FT-IR and NMR data were collected of the respected compound for characterization.

Yield = 68 %; Molecular formula = C15H22 N2S, m.p. = 124-130˚C, FT-IR (ATR) : ν(cm-1) 3431br, 3214br (N-H), 3040w (Ar, C-H), 2930w, 2850w (C-H), 1617s (C=S), 1535s (NCN), 1448s (CSasy); 1H-NMR(300 MHz,DMSO-d6) δ (ppm) = 1.22 (p, 4H, CH2), 1.64 (q, 2H, CH2), 1.89 (q, 2H, CH2), 2.13 (s, 3H, Me), 2.23 (s, 3H, Me), 2.50 (s, 2H, CH2 ), 3.17 (d, 1H, CH), 4.11 (s, 1H, NH ), 8.11 (br, 1H, NH), 6.96, 6.99, 7.03, 7.09, 7.11 (m, m, m, m, s, 3H,Ph); 13C-NMR (75 MHz, DMSO-d6) δ (ppm) = 18.0 (2C, CH2), 21.0 (1C, CH2), 25.1 (2C, Me), 32.4 (2C, Me), 52.9 (1C, CH2) (Cyclohexyl Carbons) 127.1, 128.1, 131.4, 134.7, 135.8 (6C, Ph), 180.3 (C=S).

**1-(4-Chlorophenyl)-3-cyclohexylthiourea, 4**

cyclohexylisothiocyanate (1mL, 7.05mmol) and4-chloroaniline (0.74mL, 8.3mmol) in 20 mL acetone were treated as discussed above. Colorless crystals of the desired compound 4 were obtained in the same solvent at ambient temperature.

Yield = 90%; Molecular formula = C13H17 ClN2S; m.p. = 113-118˚C; FT-IR (ATR 400-4000 cm-1) ν (cm-1) = 3929br, 3777 (N-H), 2663w (C-H), 1883s (C=C), 1718s (NCN), 1597s (CSasy); 1H-NMR (300 MHz, DMSO-d6) δ (ppm) = 1.25 (d, 2H, CH2), 1.58 (qu, 4H, CH2), 1.66 (qu,4H, CH2), 3.80 (qu,1H, CH) 4.09 (s, 1H, NH ), 9.39 (br, 1H, NH), 7.30, 7.53 7.60 (d, d, d 4H,Ph); 13C-NMR (75 MHz, DMSO-d6) δ (ppm) = 23.1, 24.9, 25.6, 32.3 32.9, 52.5, (6C, CycloHexyl), 124.5, 127.8, 128.5, 139.1 (6C, Ph), 179.6 (C=S).

**1-(3-methylpyridin-2-yl)-3-phenylthiourea, 5**

In the similar manner as described above by reacting 2-amino-3-methylpyridine (0.9 g, 8.3 mmol) with phenylisothiocyante (1 mL, 8.3 mmol), the product 5 was obtained as colorless crystalline solid by recrystallization from ethanol. In the whole reaction process the reaction was monitored by TLC until single spot product was formed.

Yield = 71 %; Molecular formula = C13H13 N3S; m.p. = 94-97˚C; FT-IR (ATR) : ν(cm-1) = 3930, 3398br (NH), 2772w (C-H), 1630s (C=S), 1360s (NCN), 1418s (CSasy); 1H-NMR(300 MHz, DMSO- d6) δ (ppm) = 2.36 (3H, Me),7.08, 7.10, 7.22, 7.68, 8.23, (m, m, m, m,8H, Ar), 9.05 (br, 1H, NH), 13.68 (br, 1H, NH);13C-NMR (75 MHz, DMSO- d6) δ (ppm) = 17.0 (1C, Me), 119.5 (2C, CH), 122.2 (1C, CH), 124.6 (2C, CH), 125.9 (1C, C-CH), 128.9 (1C, CH), 139.3 (1C CH), 140.7(1C C-CH3), 143.6 (1C, C-N), 151.8 (1C-NH), 178.7 (C=S).

**1-(3-chlorophenyl)-3-phenylthiourea, 6**

The derivative 6 was obtained as colorless solid by treating 3-chloroaniline (0.8 mL, 8.3 mmol) and phenylisothiocyanate (1.0 mL, 8.3 mmol) in the same manner as discussed above. Crystals were obtained in ethanol at room temperature by slow evaporation method.

Yield = 71 %; Molecular formula = C13H11ClN2S; m. p. = 95-99˚C; FT-IR (ATR): ν(cm-1) = 3768-3180br (N-H), 2785w (C-H), 1630s (C=S), 1589s (NCN), 1466s (CSasy);1H-NMR (300 MHz,DMSO-d6) δ (ppm) =7.12, 7.32, 7.40, 7.72, 7.8, 9.88, 9.98 (m, m, m, m, m, m, s, 9H, 2Ph), 2.51 (s, 2H, NH);13C-NMR(75 MHz,DMSO-d6) δ (ppm) = 122.3 (2C, CH), 123.4 (IC, CH), 124.3 (2C, CH), 125.2 (1C, CH), 128.9 (2C, CH), 130.4 (1C, CH), 132.6 (1C, CH), 132.9 (1C, C-Cl), 139.6 (1C, C-NH, Ph),180.1 (C=S).


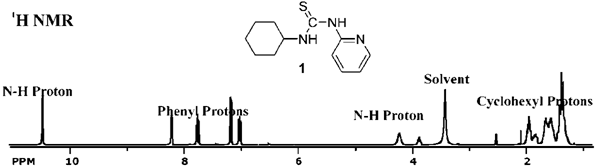


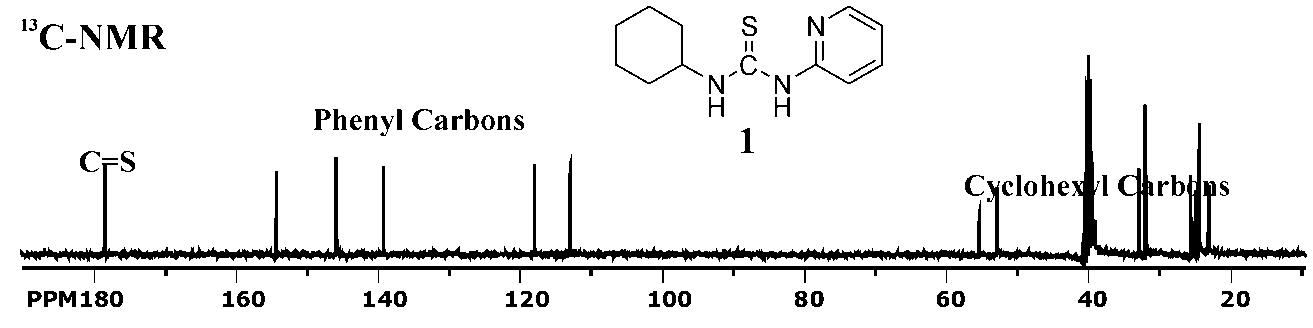


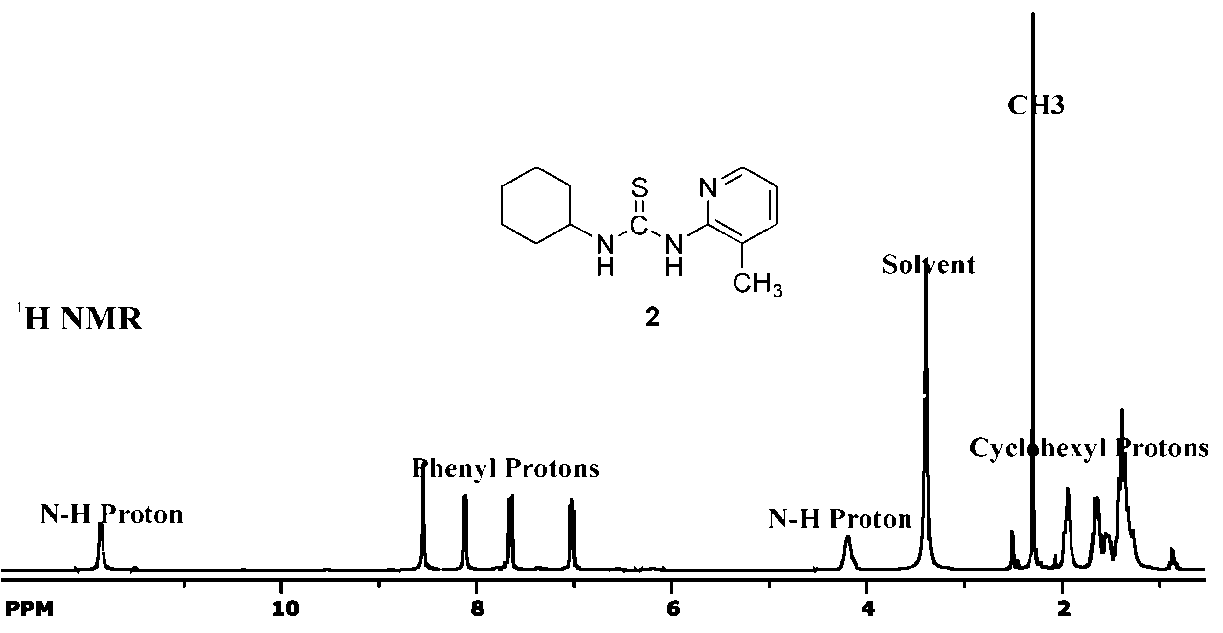


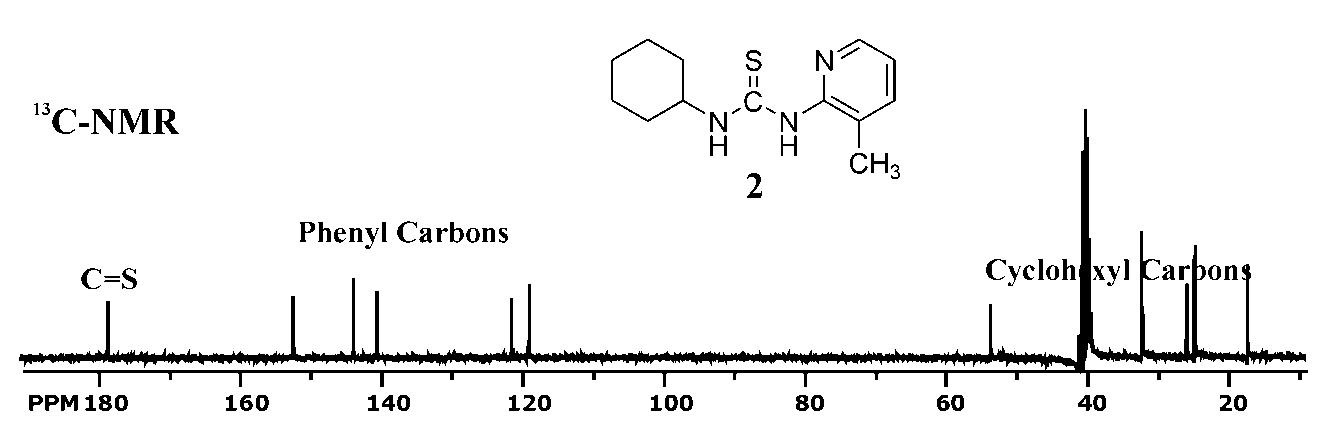


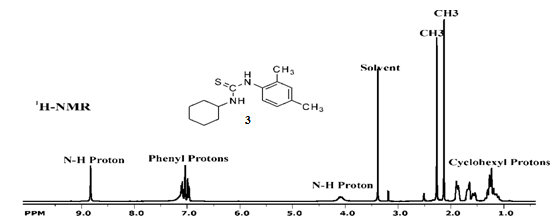


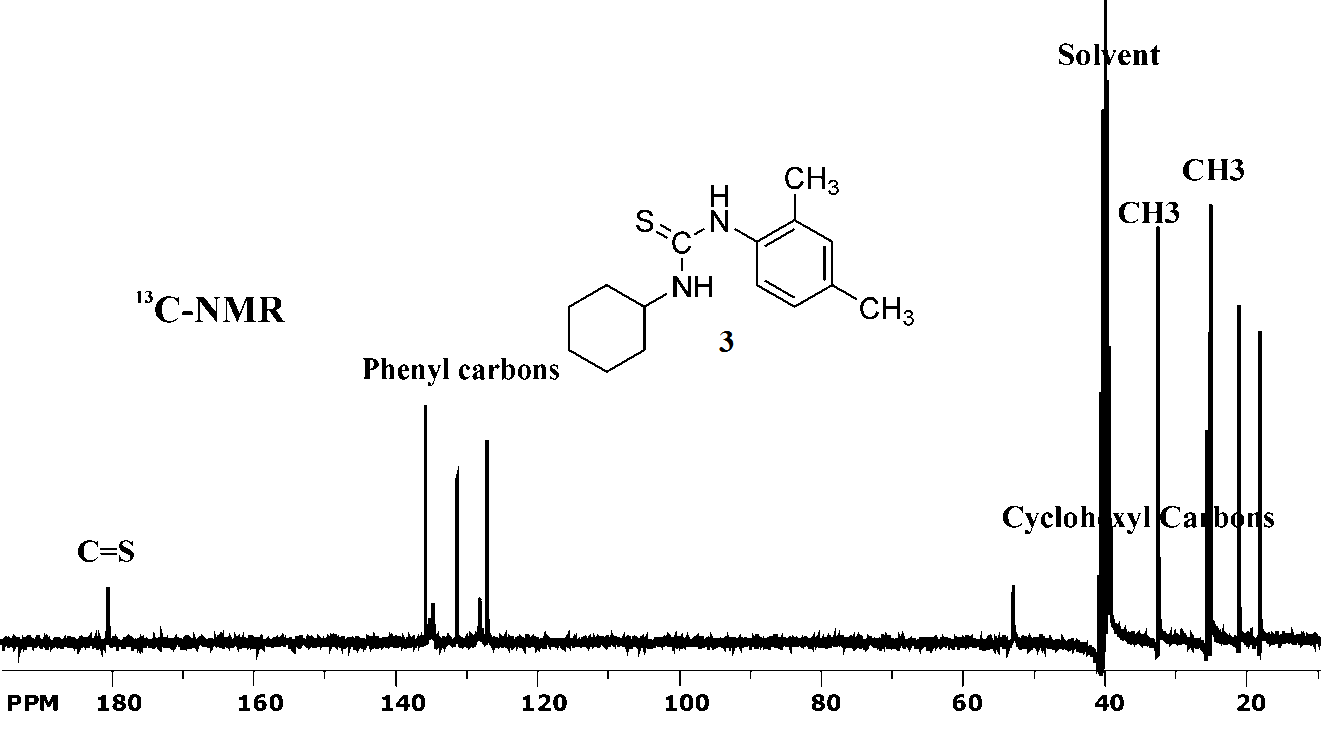


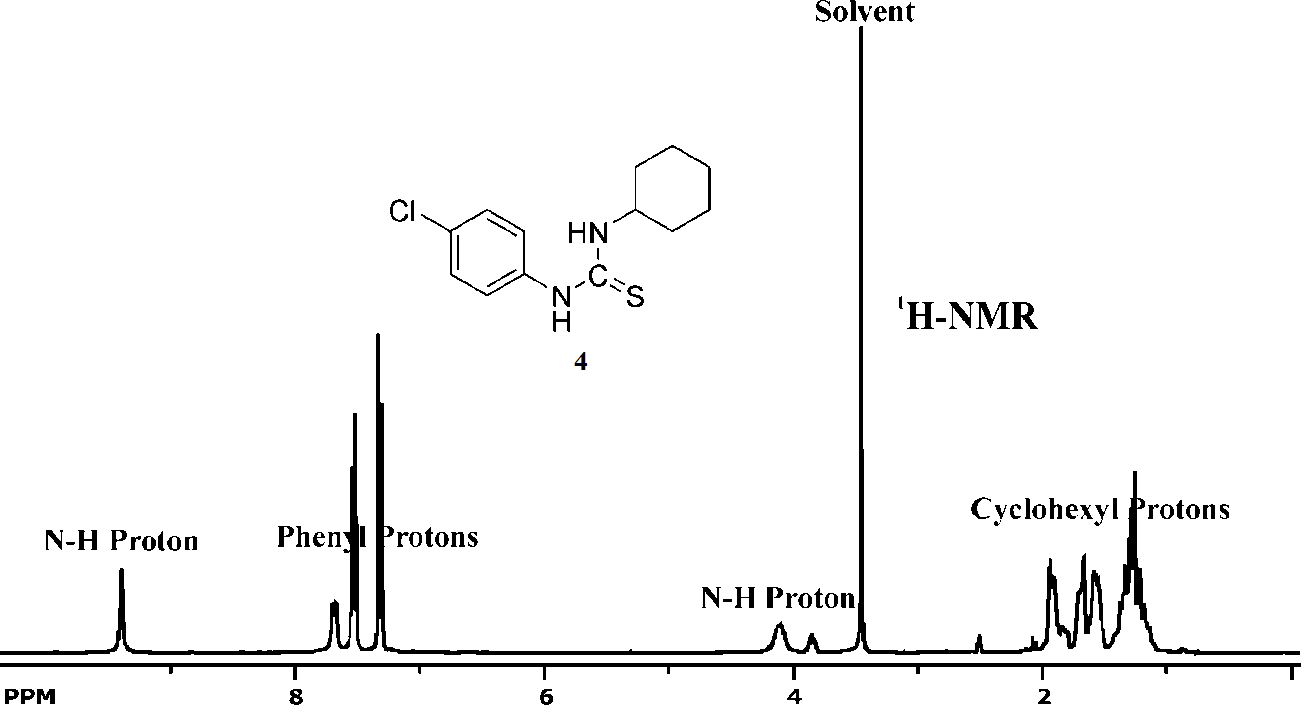


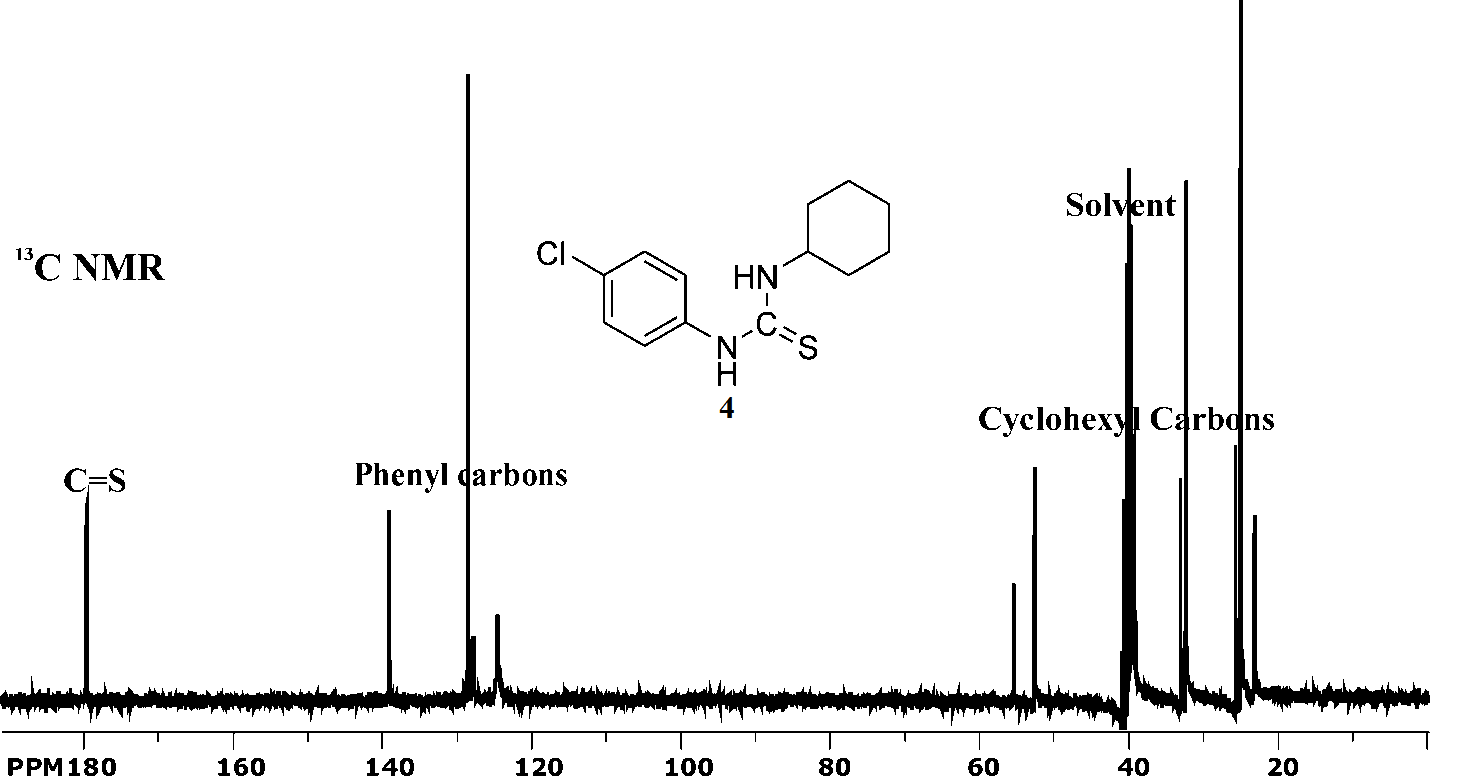


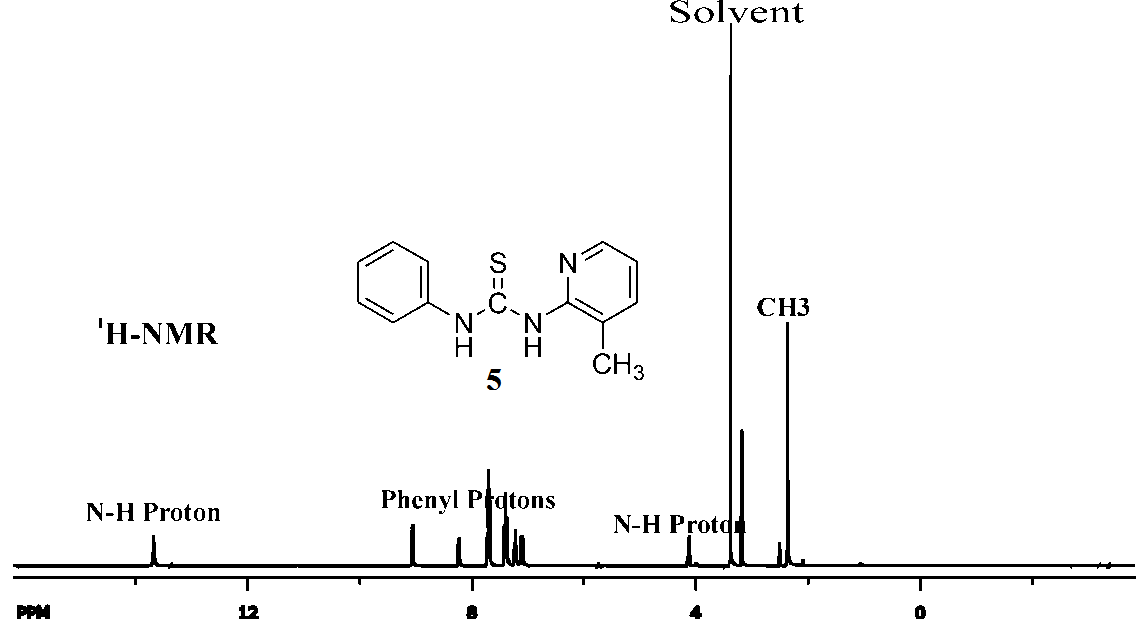


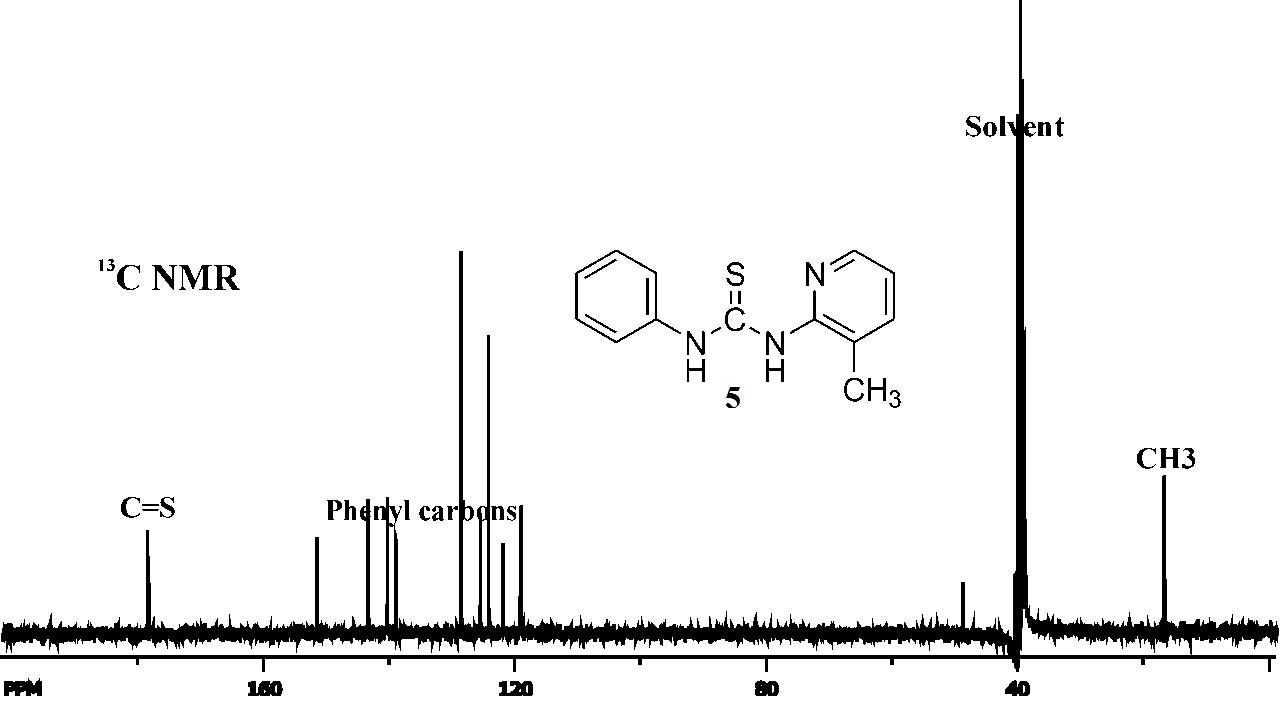


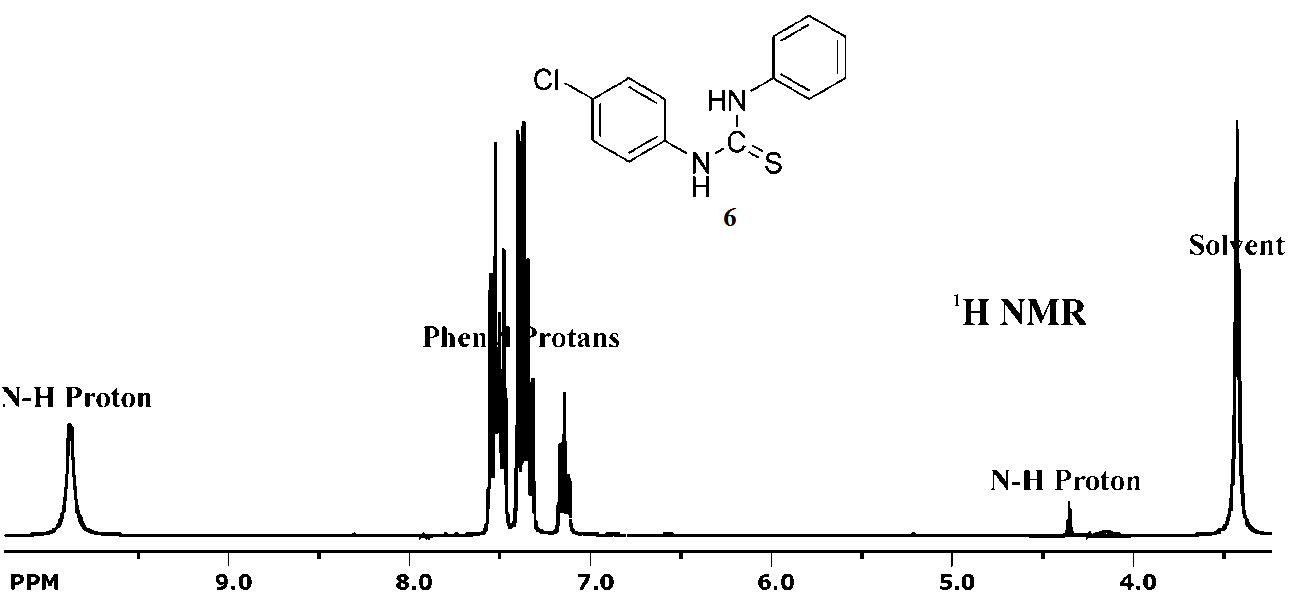


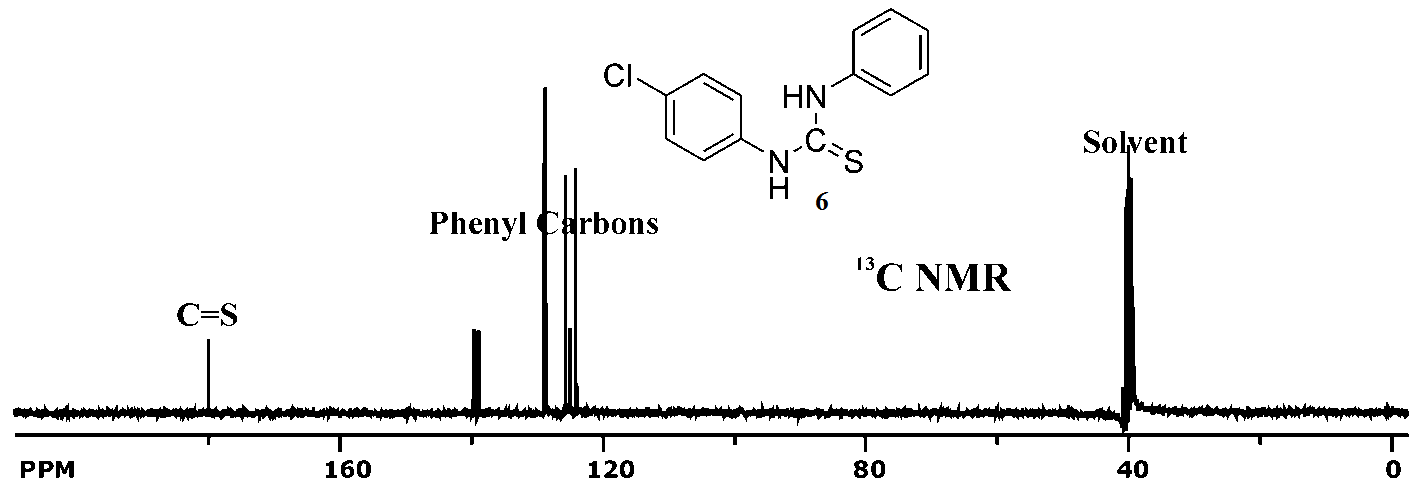


**Figure S1:** ^1^HNMR and ^13^C NMR spectra of compounds **1-6**.
